# Cytosine-to-Uracil Deamination by SssI DNA Methyltransferase

**DOI:** 10.1371/journal.pone.0079003

**Published:** 2013-10-21

**Authors:** Ildikó Stier, Antal Kiss

**Affiliations:** Institute of Biochemistry, Biological Research Center of the Hungarian Academy of Sciences, Szeged, Hungary; University of Massachusetts Medical School, United States of America

## Abstract

The prokaryotic DNA(cytosine-5)methyltransferase M.SssI shares the specificity of eukaryotic DNA methyltransferases (CG) and is an important model and experimental tool in the study of eukaryotic DNA methylation. Previously, M.SssI was shown to be able to catalyze deamination of the target cytosine to uracil if the methyl donor S-adenosyl-methionine (SAM) was missing from the reaction. To test whether this side-activity of the enzyme can be used to distinguish between unmethylated and C5-methylated cytosines in CG dinucleotides, we re-investigated, using a sensitive genetic reversion assay, the cytosine deaminase activity of M.SssI. Confirming previous results we showed that M.SssI can deaminate cytosine to uracil in a slow reaction in the absence of SAM and that the rate of this reaction can be increased by the SAM analogue 5’-amino-5’-deoxyadenosine. We could not detect M.SssI-catalyzed deamination of C5-methylcytosine (^m5^C). We found conditions where the rate of M.SssI mediated C-to-U deamination was at least 100-fold higher than the rate of ^m5^C-to-T conversion. Although this difference in reactivities suggests that the enzyme could be used to identify C5-methylated cytosines in the epigenetically important CG dinucleotides, the rate of M.SssI mediated cytosine deamination is too low to become an enzymatic alternative to the bisulfite reaction. Amino acid replacements in the presumed SAM binding pocket of M.SssI (F17S and G19D) resulted in greatly reduced methyltransferase activity. The G19D variant showed cytosine deaminase activity in *E. coli*, at physiological SAM concentrations. Interestingly, the C-to-U deaminase activity was also detectable in an *E. coli ung* ^*+*^ host proficient in uracil excision repair.

## Introduction

DNA (cytosine-5) methylation is catalyzed by C5-methyltransferases (C5-MTase), which transfer a methyl group from the methyl donor S-adenosyl-methionine (SAM) onto carbon 5 of cytosines in specific nucleotide sequences. Eukaryotic and prokaryotic C5-MTases share amino acid sequence similarity and are thought to function by the same catalytic mechanism [1]. 

 Cytosine and especially 5-methylcytosine (^m5^C) are chemically less stable than the other nucleobases. Cytosine deaminates, in a hydrolytic reaction, to uracil, and ^m5^C deaminates to thymine. The rate of spontaneous C-to-U deamination in double-stranded DNA, under physiological conditions, was found to be 2.6 - 7 x 10^-13^/s [2–4], whereas the deamination rate of ^m5^C was, under the same conditions, higher: 5.8 x 10^-13^/s [3] and 1.5 x 10^-11^/s [4].

 It was observed that the CCGG-specific prokaryotic C5-MTase M.HpaII can catalyze conversion of the target cytosine to uracil when the methyl donor SAM is missing from the reaction [5]. This enzymatic deamination is much slower than the M.HpaII-catalyzed methyltransferase reaction and is thought to be dependent on the formation of an unstable 5,6-dihydrocytosine intermediate, which can undergo hydrolytic deamination [5–7]. Subsequently, a few other prokaryotic C5-MTases [7–13] as well as the catalytic domain of the mammalian C5-MTase Dnmt3a [13], were also shown to be able to catalyze C-to-U deamination. However, this side activity does not appear to be a general feature of all C5-MTases [12].

The prokaryotic C5-MTase M.SssI shares the specificity of mammalian MTases (CG) [14], and is therefore a valuable experimental tool in the study of eukaryotic DNA methylation. M.SssI consists of 386 amino acids, contains all conserved sequence motifs of C5-MTases and probably has the same fold as other prokaryotic C5-MTases [15]. 

The possibility to use M.SssI as a CG-specific cytosine deaminase would greatly increase the value of this enzyme in epigenetics research. However, the reports in the literature on the deaminase ability of M.SssI are controversial. Some results showed that M.SssI can deaminate cytosine [7,10] or even ^m5^C [13], whereas another study did not find evidence for M.SssI-mediated cytosine deamination [4]. 

 Here we re-investigated the C-to-U and the ^m5^C-to-T deamination activity of M.SssI. Using a genetic assay, we could demonstrate slow M.SssI-catalyzed C-to-U deamination *in vitro*, in the absence of SAM. The rate of the *in vitro* reaction could be increased by 5’-amino-5’-deoxyadenosine. Under conditions where deamination of cytosine was enhanced almost 100-fold by M.SssI and 5’-amino-5’-deoxyadenosine, we could not detect M.SssI-catalyzed deamination of 5-methylcytosine. We constructed a mutant M.SssI, which showed cytosine deaminase activity in *E. coli*, at physiological concentrations of SAM. 

## Materials and Methods

### Strains, Plasmids and Growth Conditions

The following *E. coli* strains were used: ER1821 F^-^ *glnV44 e14* ^*-*^(*McrA* ^*-*^) *rfbD1? relA1? endA1 spoT1? thi-1* Δ(*mcrC-mrr*)*114::IS10* [16], DH10B F^−^ *endA1 recA1 galE15 galK16 nupG rpsL* Δ*lacX74* [17], ER2357 [endA1 *thi*-1 supE44 *mcr*-67 *ung*-1 *dut* Δ(argF-*lac*)U169 Δ(*mcr*C-*mrr*)114::IS10 recA1 F4 proAB lacIq ZΔM15 *zzf*::Tn10(Tet^R^)], DH5α F- Φ80*lac*ZΔM15 Δ(lacZYA-argF) U169 recA1 endA1 hsdR17 (rK-, mK+) phoA supE44 λ- *thi*-1 gyrA96 relA1[18]. *E. coli* ER2357-kanS and DH10B-kanS carry the inactive kanamycin resistance gene of pUP41 (see below) integrated into the bacterial chromosome. To construct the strains, the 894 bp BstBI-DraI fragment of pUP41 containing the *kanS* allele was cloned between the BstBI and PmeI sites of the plasmid pMS26 [19], and subsequently inserted into the ER2357 and DH10B chromosome using the method described in [19]. 

Plasmid pUP41 (Ap^R^ Kn^S^) carries an inactive allele of the Tn5 kanamycin resistance gene, which can revert to Kn^R^ phenotype by a C-to-T mutation [20]. 

Plasmid pBHNS-MSssI carries the gene of C-terminally His-tagged M.SssI [21] cloned in pBAD24 (Ap^R^) [22]. The *sssIM* allele cloned in pBHNS-MSssI was considered as wild-type for this work. Plasmids pBHNS-MSssI(F17S) and pBHNS-MSssI(G19D) encode mutant variants of M.SssI, and were created from pBHNS-MSssI by site-directed mutagenesis [23]. Plasmid pSTC-MSssI (former name pSTB-MSssI) [24] contains the gene of M.SssI (WT) in the pSC101-based plasmid vector pST76-C (Cm^R^) [25] characterized by heat-sensitive replication. Plasmids pSTdC-MSssI, pSTdC-MSssI(F17S) and pSTdC-MSssI(G19D) are derivatives of pSTC-MSssI and carry the genes of untagged WT or mutant M.SssI as indicated. The vector part in the latter three plasmids differs from that of pSTC-MSssI by a 98 bp deletion between the AseI and PstI sites. The deletion was introduced to facilitate subsequent cloning steps. In all plasmids carrying the *sssIM* gene, M.SssI expression was under the control of the arabinose P_BAD_ promoter and the AraC protein [22]. All M.SssI variants used in this work carried the C368A replacement, which does not affect MTase activity of WT M.SssI [21].

Bacteria were routinely grown in LB medium [26] at 30 or 37 °C. For M.SssI expression, cells containing plasmids with the M.SssI gene were grown at 30°C, and M.SssI production was induced by adding 0.1% arabinose to the medium. SOC/SOB medium was used for preparation of electrocompetent cells and TB medium [26] to grow *E. coli* for purification of M.SssI. Ampicillin (Ap), kanamycin (Kn) and chloramphenicol (Cm) were used at 100, 50 and 25 μg/ml concentration, respectively.

### Oligonucleotides

The deoxyoligonucleotides AK233 (GTA TTT GAA GCT TCT GCT GGA ATT GG) and AK234 (GAA GCT TTT GCT GAC ATT GGT GCT CAA AG) synthesized in this institute were used to introduce the F17S and G19D replacements, respectively. AK233 and AK234 represent the coding strand of the *sssIM* gene with the nucleotides corresponding to the mutations underlined.

### Purification of M.SssI

His-tagged wild-type and mutant M.SssI variants were purified from *E. coli* DH10B or ER1821 cells harboring pBHNS-MSssI, pBHNS-MSssI(F17S) or pBHNS-MSssI(G19D) and grown in TB/Ap. At a cell density of OD_600_~0.5, 0.1% arabinose was added to induce MTase production, and growth was continued at 30°C for 4 - 6 hours. Cells from 400 ml culture were harvested, resuspended in a buffer containing 50 mM Tris-HCl pH 8.0, 1mM EDTA, 10mM β-mercaptoethanol, 5% glycerol and disrupted by sonication. Cell debris was removed by centrifugation, and the supernatant was loaded onto a heparin-agarose column. Proteins were eluted with a 0 - 1M NaCl gradient in a buffer containing 50 mM Tris-HCl pH 8.0 and 5% glycerol. Peak active fractions were pooled, diluted two-fold with Ni-agarose equilibration buffer (50 mM Tris-HCl pH 7.8, 0.5 M NaCl and 1 mM imidazole) and loaded onto a Ni-agarose column (His-Select, Sigma) equilibrated with the same buffer. Proteins were eluted using a buffer containing 50 mM Tris-HCl pH 7.5, 0.5 M NaCl and 250 mM imidazole. M.SssI-containing fractions were concentrated by dialysis against storage buffer (50 mM Tris-HCl pH 7.5, 100 mM NaCl, 1 mM EDTA, 10 mM β-mercaptoethanol and 50% glycerol), and stored at -20°C. 

In some cases the heparin-agarose step was omitted and the diluted crude extract was loaded directly onto the Ni-agarose column. Purity of enzyme preparations varied between 60-80% as determined by SDS-polyacrylamide gel electrophoresis.

### DNA Methyltransferase Reaction

M.SssI activity was routinely estimated by restriction protection assay. Samples from a serial dilution of M.SssI were incubated with 0.2 - 0.5 µg plasmid or lambda phage DNA in M.SssI reaction buffer (50 mM Tris-HCl pH 8.5, 50 mM NaCl, 10 mM EDTA, 5 mM DTT containing 350 µg/ml bovine serum albumin) containing 160 μM SAM (New England Biolabs) at 30°C for one hour. After the reaction the DNA was purified by phenol/chloroform extraction and ethanol precipitation. The precipitated DNA was dissolved, digested with Hin6I restriction enzyme and analyzed by agarose gel electrophoresis. In some cases phenol/chloroform extraction was omitted and M.SssI was inactivated by heat treatment (60°C 20 min) before adding Hin6I. 

### Cytosine Deamination *in vitro*

Plasmid pUP41 (70 - 110 ng) was incubated with purified M.SssI in M.SssI reaction buffer (see above) in 50 µl at 30°C for 4 h or as shown at the particular experiment. Under these conditions the concentration of double-stranded CG sites in the reaction was ~0.18 - 0.27 µM. M.SssI was used at concentrations indicated in the text. Some deamination reactions contained SAM, sinefungin (Ili Lilly or Sigma) or 5’-amino-5’-deoxyadenosine (Sigma) at concentrations indicated in the text. After the incubation, the reactions were stopped with 0.5% SDS, and the DNA was purified by phenol/chloroform extraction and ethanol precipitation. The precipitated DNA was dissolved in TE buffer (10 mM Tris-HCl pH 8.0, 1 mM EDTA), and was used to transform *E. coli* ER2357 *ung* or DH10B *ung* ^*+*^ cells by electroporation. Appropriate dilutions of the bacterial suspension were spread on Ap and Kn plates to determine the number of Ap^R^ and Kn^R^ transformants. 

For testing deamination of C5-methylcytosine, CG-specifically methylated pUP41 was prepared either *in vivo*, in DH10B cells that also contained pSTC-MSssI and were grown in the presence of 0.1% arabinose, or *in vitro* using purified M.SssI and SAM. In either case complete methylation was verified by Hin6I digestion. 

### Cytosine Deamination *in vivo*

M.SssI-mediated cytosine deamination *in vivo* was studied by two methods. In the simpler reversion test, *E. coli* ER2357-kanS *ung* and DH10B-kanS *ung* ^+^ harboring one of the plasmids pBHNS-MSssI, pBHNS-MSssI(F17S) or pBHNS-MSssI(G19D) were grown in LB/Ap/0.2% glucose. The growth medium contained glucose to repress M.SssI expression. Cells from this culture were sedimented by centrifugation, washed in glucose-free LB, and used to inoculate fresh LB/Ap/0.1% arabinose medium. After 4 h growth at 30°C, frequency of Kn^R^ revertants was determined by spreading aliquots of serial dilutions on Kn and Ap plates. 

The rate of C-to-U deamination *in vivo* was determined by the fluctuation test. *E. coli* ER2357 *ung* and DH10B *ung* ^*+*^ harboring pUP41 were transformed with pSTdC-MSssI, pSTdC-MSssI(F17S) or pSTdC-MSssI(G19D). Ap^R^ Cm^R^ transformants were grown in the presence of 0.2% glucose to repress M.SssI expression. Cells from overnight cultures were centrifuged, resuspended in glucose-free LB, and were used to inoculate 10 parallel 1 ml cultures in LB/Ap/Cm/0.1% arabinose. After 24 h growth at 30°C, the number of Kn^R^ and Ap^R^ colonies was determined as described above. The reversion rate was calculated by the on-line FALCOR program using the Ma-Sandri-Sarkar Maximum Likelihood Estimator method [27]. 

### Other Methods

DNA cloning, PCR reactions, agarose gel electrophoresis of DNA and polyacrylamide gel electrophoresis of protein samples were done by standard methods [26]. Enzymes were purchased from Fermentas (Thermo Scientific) or from New England Biolabs. Statistical evaluation of data was performed with the GraphPad Prism software package (GraphPad Software Inc.). P values were calculated by one-way ANOVA test using GraphPad Prism. 

## Results

### Cytosine Deamination *in vitro*

To detect C-to-U deamination by M.SssI, we used a genetic reversion assay developed by Bhagwat and coworkers [20]. This assay employs the Ap^R^ plasmid pUP41 carrying an inactive, mutant allele of the kanamycin resistance gene of the transposon Tn5. The mutant codon resulting in L94P substitution and kanamycin sensitivity is located within a SmaI restriction site CCCGGG. Conversion of the underlined cytosine to thymine reverts the amino acid substitution to wild-type Leu94 and restores kanamycin resistance. Because the underlined cytosine is in a CG dinucleotide, the substrate site for SssI DNA methyltransferase [14], pUP41 can be used to assay M.SssI-catalyzed cytosine deamination. Deamination of cytosine first creates a U:G mismatch, which – if left unrepaired – is converted to C-to-T mutation after DNA replication. The 6804 bp pUP41 plasmid contains 534 CG dinucleotides. Reversion to kanamycin resistance by cytosine deamination as described above eliminates the SmaI site and creates a new MvaI site (CCWGG) [10] and Figure S1.

**Figure 1:**
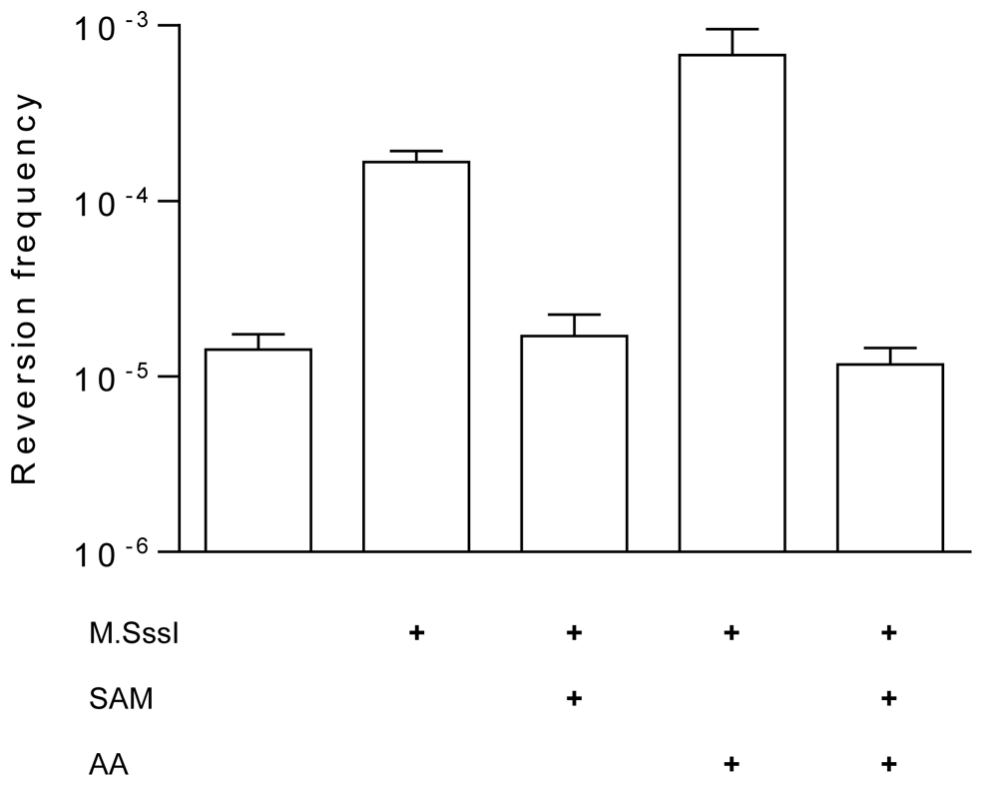
Cytosine deamination by M.SssI *in* *vitro*. pUP41 (Ap^R^, Kn^S^) plasmid DNA was incubated with or without wild-type M.SssI (2-fold molar excess relative to CG sites) at 30°C for 4 h, and frequency of C-to-U deamination was determined by scoring the numbers of Kn^R^ and Ap^R^ transformants in *E. coli* ER2357 *ung* strain. SAM (160 µM) and 5’-amino-5’-deoxyadenosine (AA, 250 μM) were added to samples as indicated. Error bars represent standard error of the mean of at least three independent experiments (p<0.01).

His-tagged M.SssI was purified as described in Materials and Methods. Plasmid pUP41 was incubated with M.SssI in the absence of the methyl donor SAM, then introduced by electroporation into the *E.coli* ER2357 *ung* strain deficient in the repair of uracil containing DNA. The frequency of C-to-U conversions was derived from the ratio of the Kn^R^ and Ap^R^ transformants. Preliminary experiments testing the conditions of M.SssI-mediated cytosine deamination indicated that the number of Kn^R^ revertants reached maximal level at a ~two-fold excess of the enzyme over CG sites in the plasmid and after 4 h incubation at 30°C (not shown). The influence of the incubation temperature was not tested specifically for the deamination activity. Previously we found that the MTase activity of M.SssI was, in vitro as well as *in vivo*, higher at 30°C than at 37°C (unpublished results). Assuming that the deamination and methyltransferase activities of M.SssI have the same optimal temperature, 30°C was used throughout this work. In most experiments ~twofold MTase/CG site ratio and 4 h incubation time were used as standard conditions. Under these conditions, the reversion frequency varied between 10^-4^ and 10^-5^ for the plasmid incubated without the enzyme. M.SssI increased the reversion frequency ~10-fold. Addition of SAM lowered the reversion frequency back to the level of the untreated plasmid (Figure 1). No revertants were obtained when the M.SssI-treated DNA was transformed into the Ung^+^ *E. coli* host DH10B indicating that reversion to Kn^R^ phenotype went through the C-to-U-to-T pathway (not shown). Plasmids were isolated from some of the Kn^R^ clones and the disappearance of the SmaI site and the concomitant appearance of a new MvaI site was verified by restriction digestion (Figure S1). 

### SAM Binding Pocket Mutants, Cytosine Deamination *in vivo*

Conserved block I characterized by the **F**X**G**X**G** sequence motif is part of the SAM binding pocket in C5-MTases [1,28,29]. To be able to investigate cytosine deamination by the CCGG-specific C5-MTase M.HpaII *in vivo*, in the presence of SAM, Jones and coworkers introduced substitutions (F38S and G40D) in the SAM binding pocket of M.HpaII [30]. 

**Figure 2:**
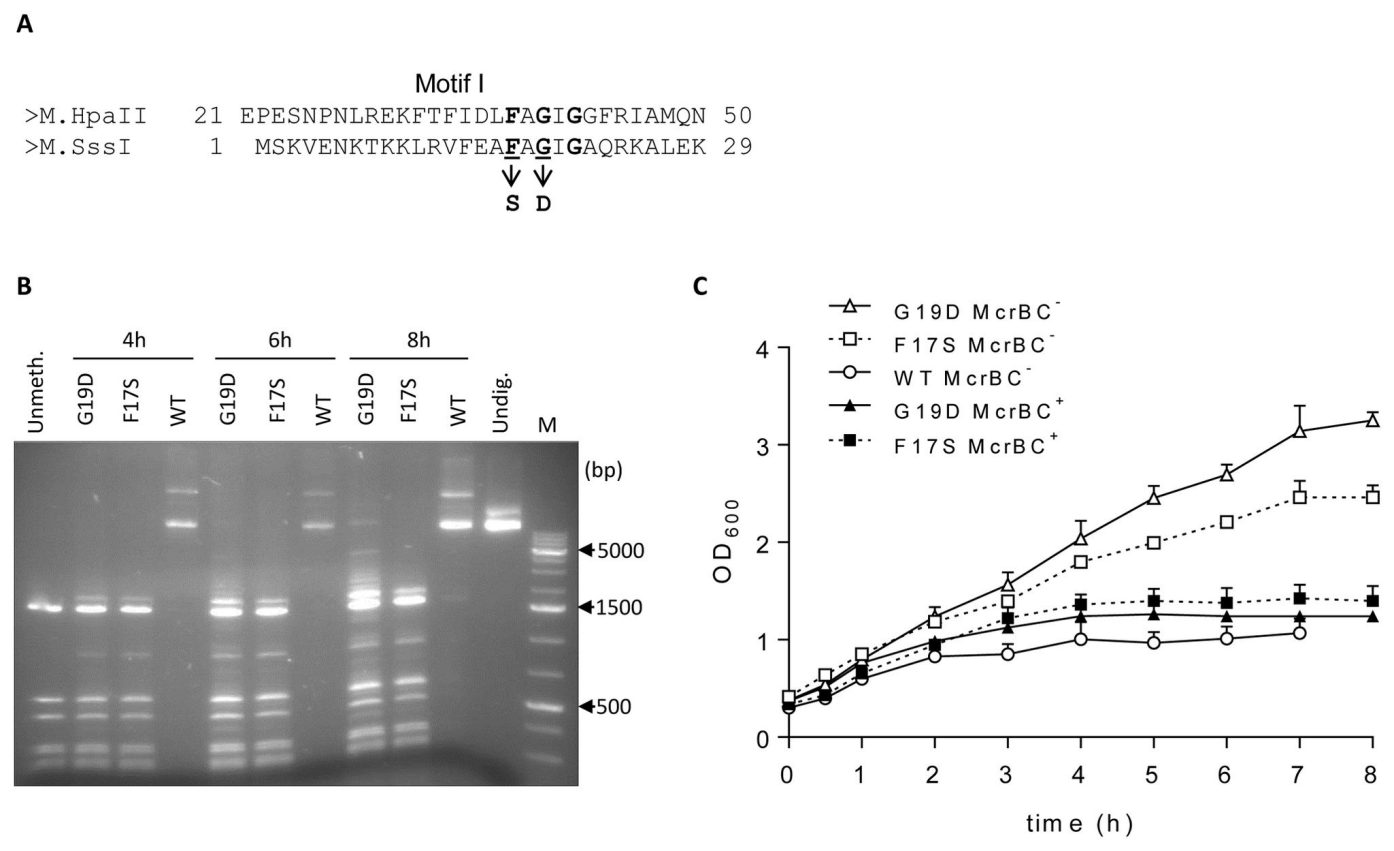
DNA methyltransferase activity of the F17S and G19D M.SssI mutants. (**A**) Amino acid sequence alignment between segments of M.HpaII and M.SssI. Conserved residues of the FXGXG motif are in bold. The F17S and G19D substitutions are indicated below the sequence. (**B**) Hin6I digestion of plasmids pBHNS-MSssI, pBHNS-MSssI(F17S) and pBHNS-MSssI(G19D) encoding WT or mutant M.SssI variants, respectively as indicated above the lanes. Plasmids were isolated from cultures grown for 4-6-8 h in the presence of arabinose to induce M.SssI expression. Resistance to Hin6I (recognition sequence GCGC) indicates M.SssI-specific methylation (R. Kazlauskiene, cited in REBASE [45]. Lane Unmeth., unmethylated pBHNS-MSssI(G19D) isolated from cells grown in the presence of glucose; Lane Undig., undigested pBHNS-MSssI; M, molecular weight marker (GeneRuler 1 kb DNA Ladder, Fermentas). (**C**) Effect of WT and mutant M.SssI production on growth of *E. coli* *mcrBC* and *mcrBC* ^+^ hosts. DH10B *mcrBC* contained pBHNS-MSssI, pBHNS-MSssI(F17S) or pBHNS-MSssI(G19D) as indicated. DH5α *mcrBC* ^*+*^ contained pBHNS-MSssI(F17S) or pBHNS-MSssI(G19D). Bacteria were grown in LB/Ap medium at 30°C. MTase expression was induced at time 0 by arabinose. Error bars represent standard error of the mean of three independent experiments.

To study cytosine deamination by M.SssI *in vivo*, we used the same strategy. In M.SssI, the amino acids corresponding to F38 and G40 of M.HpaII are F17 and G19 (Figure 2A). The F17S and G19D substitutions were created by site-directed mutagenesis. Two types of plasmids were constructed. To obtain plasmids compatible with pUP41, the WT and the mutant *sssIM* alleles were transferred into the plasmid vector pST76-C [25] to yield pSTdC-MSssI, pSTdC-MSssI(F17S) and pSTdC-MSssI(G19D). For high level expression of the M.SssI variants, the mutations leading to the F17S and G19D substitutions were transferred into the ColE1-based, high copy number plasmid pBHNS-MSssI to obtain pBHNS-MSssI(F17S) and pBHNS-MSssI(G19D). In both types of plasmids, transcription of the *sssIM* gene was inducible with arabinose. 

**Figure 3:**
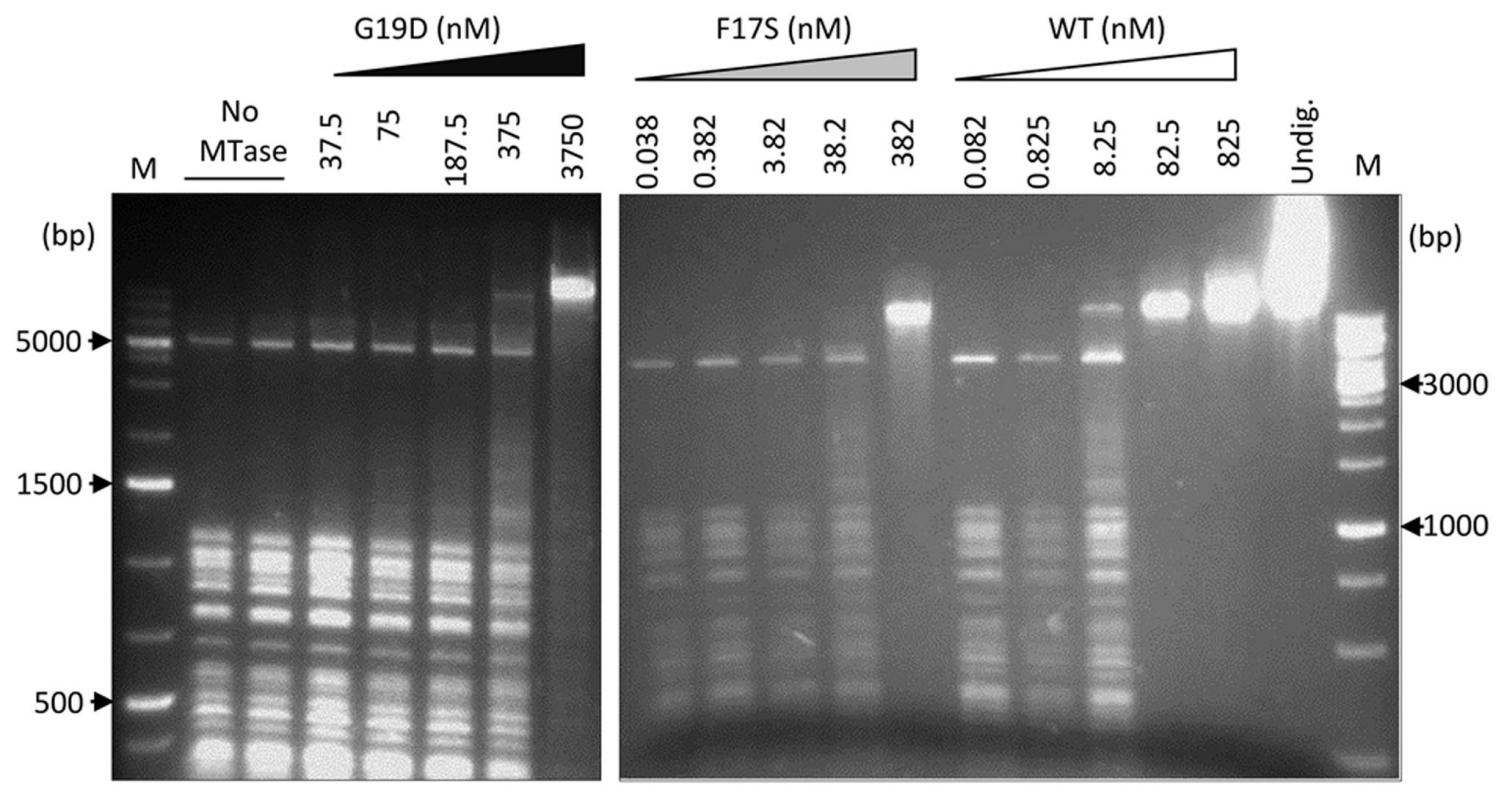
Estimation of DNA MTase activity of the F17S and G19D M.SssI mutants by restriction enzyme protection assay. Lambda phage DNA was incubated with different concentrations of WT and mutant M.SssI in the presence of SAM as described in Materials and Methods. Methylation status of the DNA was subsequently tested by digestion with the methylation sensitive restriction enzyme Hin6I, and the digestion was analyzed by agarose gel electrophoresis. Lane Undig., undigested plasmid; M, molecular weight marker (GeneRuler 1 kb Plus and GeneRuler 1 kb DNA Ladders, Fermentas). M.SssI-mediated cytosine deamination *in* *vivo* was initially investigated using a two-plasmid-system, with the *E. coli* host containing the indicator plasmid pUP41 and one of the M.SssI-expressing plasmids pSTdC-MSssI, pSTdC-MSssI(F17S) or pSTdC-MSssI(G19D). The latter plasmids have pSC101 replicon and are compatible with pUP41. We observed elevated reversion frequency to kanamycin resistance with the Ung^-^ host ER2357 expressing the G19D variant (not shown).

To test the effect of the F17S and G19D replacements on the MTase activity, DH10B cells harboring pBHNS-MSssI or its mutant derivatives were grown to mid-log phase, then arabinose was added to induce M.SssI expression. Plasmid DNA was purified from the cultures after 4, 6 and 8 h induction, digested with the methylation sensitive restriction enzyme Hin6I and analyzed by agarose gel electrophoresis. After 4 h induction, pBHNS-MSssI encoding the WT MTase was completely resistant, whereas pBHNS-MSssI(F17S) and pBHNS-MSssI(G17D) were almost fully digestible. After 6 and 8 h growth partial digestion products appeared in the digests of the mutant plasmids (Figure 2B). pBHNS-MSssI(G19D) was slightly more protected against Hin6I digestion than pBHNS-MSssI(F17S). These results showed that both mutants had drastically reduced but detectable MTase activity. The residual MTase activities were consistent with the viability of *E. coli* McrBC^*+*^ and McrBC^-^ hosts expressing the mutant enzymes. McrBC nuclease cuts DNA containing (G/A)^m5^C sites [31], thus M.SssI-specific DNA methylation should cause DNA degradation in McrBC^*+*^ hosts. As expected, expression of the mutant MTases led to growth arrest in the McrBC^*+*^ host, whereas McrBC^-^ cells continued growing after induction (Figure 2C). Comparison of the MTase activities of the purified enzymes confirmed the observations made *in vivo*: the purified F17S and G19D mutant enzymes had substantially lower MTase activity *in vitro* than the WT enzyme (Figure 3). Similar observations were made when the activities of the purified enzymes were compared using a radioactive assay measuring incorporation of ^3^H-labeled methyl groups (not shown). 

**Figure 4:**
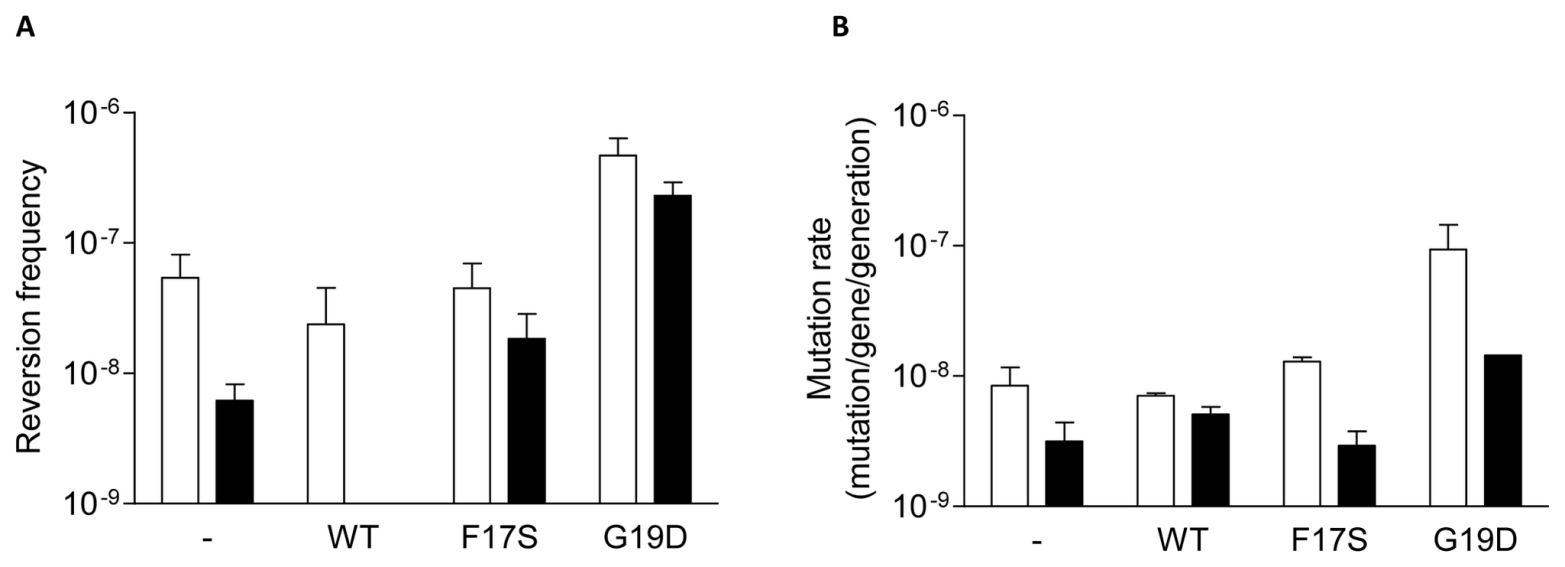
Cytosine deamination by WT and mutant (F17S and G19D) M.SssI *in vivo.* (**A**) *E. coli* ER2357-kanS *ung* and DH10B-kanS *ung* ^*+*^ harbouring pBHNS-MSssI, pBHNS-MSssI(F17S) or pBHNS-MSssI(G19D) encoding WT or mutant M.SssI were grown in the presence of arabinose to induce M.SssI expression. Frequency of Kn^R^ revertants was determined after 4 h growth. Due to poor growth after arabinose induction the reversion frequency of DH10B-kanS expressing the WT MT could not be reliably determined (Figure 4A). Empty bars, *ung-*; filled bars, *ung+*. Error bars represent standard error of the mean of three independent experiments (p<0.01). (**B**) *E. coli* ER2357 *ung* and DH10B *ung* ^*+*^ cells contained pUP41 (Ap^R^ Kn^S^) and a compatible plasmid (pSTdC-MSssI, pSTdC-MSssI(F17S) or pSTdC-MSssI(G19D) expressing WT or mutant M.SssI. Mutation rates were calculated from reversion to Kn^R^ phenotype by fluctuation tests as described in Materials and Methods. Empty bars, *ung-*; filled bars, *ung+*. Results of the following number of experiments: no MTase (3), WT and F17S (2), G19D (4). Error bars represent standard error of the mean.

Later, to eliminate any stochastic effects associated with plasmid segregation, we transferred the reporter KanS gene from pUP41 onto the chromosome of ER2357 and DH10B to obtain the strains ER2357-kanS *ung* and DH10B-kanS *ung* ^+^. Due to the site specific mechanism of the technique used [19], the two strains contained the KanS gene in the same locus of their genome. Location of the reporter gene on the chromosome had the added advantage that we could use the ColE1-based pBHNS- plasmids to introduce the *sssIM* alleles. These plasmids have much higher copy number than the pSTdC- plasmids and result in higher M.SssI concentration after arabinose induction. ER2357-kanS *ung* and DH10B-kanS *ung* ^+^ harboring pBHNS-MSssI, pBHNS-MSssI(F17S) or pBHNS-MSssI(G19D) were induced at mid-log phase with arabinose, then after 4 h growth the frequency of Kn^R^ revertants was determined as described in Materials and Methods. The Ung^-^ host not producing M.SssI showed a reversion frequency between 10^-7^ and 10^-8^, which can be considered to reflect the spontaneous C to U deamination rate under these conditions (Figure 4A). The reversion frequency in the Ung^*+*^ host lacking M.SssI was at least an order of magnitude lower than in the Ung^-^ host. Expression of the WT or the F17S mutant enzyme did not enhance the reversion frequency, whereas the G19D variant caused an ~10-fold increase relative to the spontaneous deamination frequency in the Ung^-^ as well as in the Ung^+^ host (Figure 4A). 

**Figure 5:**
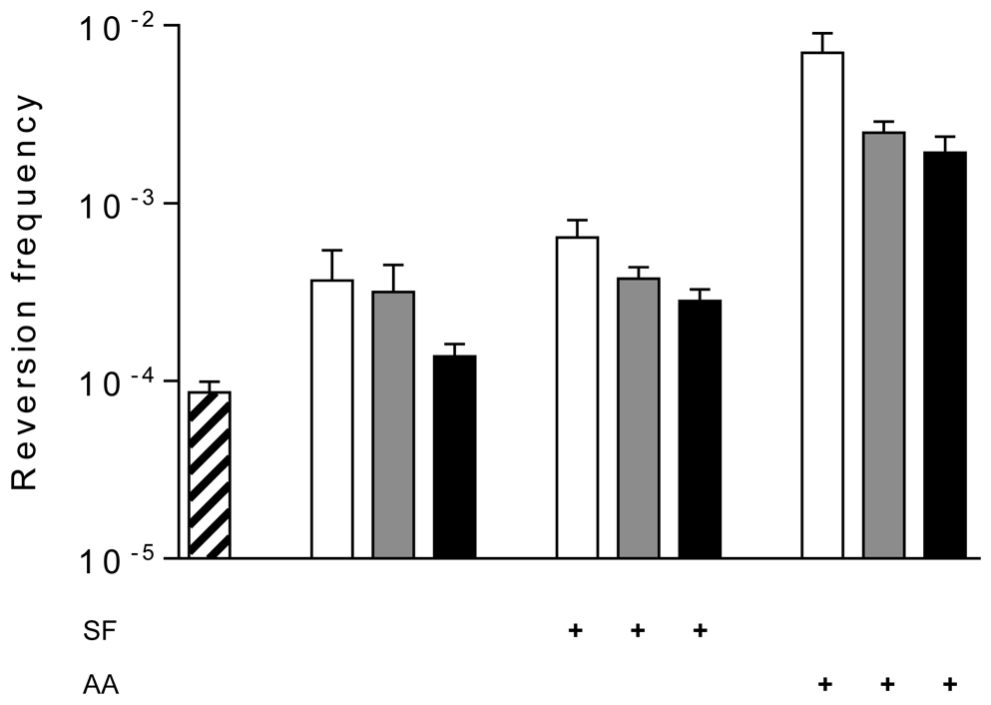
Effect of sinefungin and 5’-amino-5’-deoxyadenosine on cytosine deamination activity of M.SssI and its mutants. Plasmid pUP41 was incubated with purified M.SssI, M.SssI(F17S) or M.SssI(G19D) and frequency of Kn^R^ revertants was determined in ER2357 *ung* strain as described in Materials and Methods and legend of Figure 1. Sinefungin (SF) was used at 500 and 5’-amino-5’-deoxyadenosine (AA) at 250 µM concentration in samples indicated below the bars. Reversion frequency was derived from the ratio of the Kn^R^ and Ap^R^ transformants. Striped bar, no enzyme; empty bars, wild-type M.SssI; grey bars, M.SssI(F17S); black bars, M.SssI(G19D). Error bars represent standard error of the mean of five independent experiments (p<0.01).

 Because mutation frequencies determined by the simple reversion assay may not reliably reflect mutation rates [32], C-to-U deamination rates were also estimated by fluctuation test [27,32,33]. Results of the fluctuation tests confirmed the tendency seen in the simpler reversion tests: the G19D replacement resulted in an ~11-fold rate enhancement relative to the strain not expressing M.SssI (Figure 4B). Surprisingly, M.SssI(G19D) increased reversion also in the Ung^+^ host (Figure 4). Similar unexpected increase in the frequency of C to U conversion was observed with the HpaII MTase, and was attributed to strong binding of the MTase to the premutagenic U:G base pair and blocking repair [30]. Cytosine deamination activity of the purified F17S and G19D mutant enzymes was similar to that of the WT MTase (Figure 5). 

### Effects of SAM Analogues

 Two SAM analogues, sinefungin (SF) and 5’-amino-5’-deoxyadenosine (AA) were previously shown to promote cytosine deamination by M.HpaII, HhaI, MspI as well as by M.SssI [7,12]. It was suggested that these compounds acted by increasing protonation of cytosine C5 [7]. Another study demonstrated the same phenomenon for M.EcoRII, but provided evidence that the stimulatory effect of 5’-amino-5’-deoxyadenosine does not involve enhancing protonation of C5 [34]. We tested the effect of sinefungin and 5’-amino-5’-deoxyadenosine on the cytosine deamination activity of the F17S and G19D M.SssI mutants. Initial experiments testing concentration-dependence indicated that sinefungin and 5’-amino-5’-deoxyadenosine reached maximal effect at 500 and 250 µM, respectively (not shown). At these concentrations sinefungin led to a slight, whereas 5’-amino-5’-deoxyadenosine to a greater increase of deamination activity of the mutant enzymes. However, the rate enhancement was greatest for the WT MTase (Figure 5). The weaker stimulation of F17S and G19D by sinefungin or 5’-amino-5’-deoxyadenosine probably reflects the intended lower cofactor binding affinity of the mutant enzymes. Under the conditions used, 250 µM 5’-amino-5’-deoxyadenosine increased deamination by the WT enzyme almost 20-fold, which was an approximately 80-fold enhancement relative to the rate of the untreated plasmid (Figure 5). If SAM was present, 5’-amino-5’-deoxyadenosine had no effect (Figure 1). 

### Lack of C5-methylcytosine Deaminase Activity

**Figure 6:**
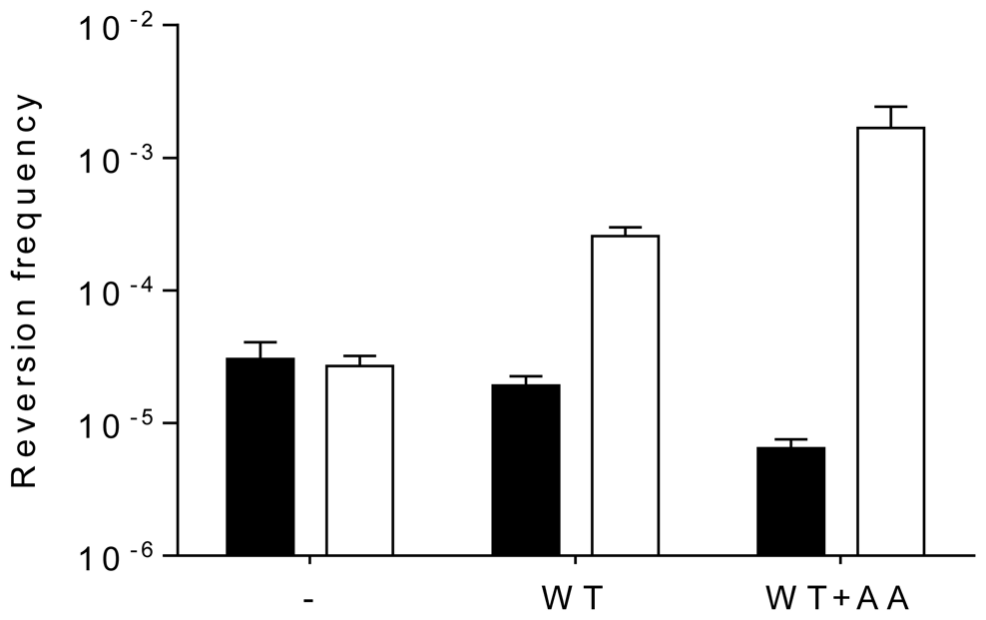
Comparison of cytosine and 5-methylcytosine deamination by M.SssI. Unmethylated and *in* *vivo* M.SssI-specifically methylated pUP41 plasmid DNA was incubated with or without M.SssI(WT) and 5’-amino-5’-deoxyadenosine as indicated using conditions described in the legend of Figure 1. Frequency of reversion to Kn^R^ phenotype was determined for unmethylated pUP41 in ER2357 *ung* (empty bars), whereas reversion frequency of methylated pUP41 was determined in DH10B *ung* ^+^ host (filled bars). Error bars represent standard error of the mean of at least three independent experiments (p<0.01).

To test whether M.SssI can deaminate C5-methylcytosine in double-stranded DNA, CG-specifically methylated pUP41was prepared as described in Materials and Methods and treated with purified M.SssI(WT) in the absence of SAM using the same conditions as described for deamination of unmethylated pUP41. The only difference was that, to minimize the possibility of scoring C-to-U deaminations as ^m5^C-to-T deamination events, reversion frequency was determined using the Ung^*+*^ host DH10B. The reversion frequencies of the untreated plasmids were the same (Figure 6). Under conditions where the reversion frequency of unmethylated pUP41 was increased ~10-fold by M.SssI and ~100-fold by the combined action of M.SssI and 5’-amino-5’-deoxyadenosine, the reversion frequency of methylated pUP41 was not increased relative to the spontaneous rate (Figure 6). This finding was in contrast with results of other investigators [13], who used a completely *in vitro* assay to detect U:G or T:G mismatches resulting from deamination of cytosines or 5-methylcytosines, respectively. They observed that M.SssI could catalyze deamination of cytosine as well as of 5-methylcytosine. Interestingly, under their assay conditions, 5-methylcytosine appeared to be a better substrate than cytosine: the deamination rate of 5-methylcytosine was higher by ~30% than that of cytosine. Moreover, C-to-U conversion required the presence of 5-aminoadenosine or sinefungin, whereas the ^m5^C-to-T reaction was detectable in the absence of these cofactor analogues (supplementary information of ref [13].. The reaction conditions used by Metivier et al. were slightly different from ours. Most notably, the pH of their reaction buffer was 7.5, the buffer contained Mg^2+^ and the samples were incubated overnight at 37°C [13]. To address the discrepancy between the results, we performed deamination reactions using conditions of the Metivier et al. study [13] (same buffer, except that protease inhibitor was not added, incubation was at 37°C for 16 h). We observed enhanced reversion rate with unmethylated pUP41, but not with methylated pUP41; the deamination rate of the methylated plasmid was even lower than in experiments with our standard conditions (not shown). 

## Discussion

Bisulfite sequencing is a widely used method to identify C5-methylated cytosines in DNA [35]. The method suffers from some limitations such as occasional incomplete conversion of cytosines to uracil or degradation of the DNA during the bisulfite treatment [36]. This study was motivated by the interest to develop an enzymatic alternative to bisulfite-mediated C-to-U conversion for cytosines located in CG dinucleotides, which are the predominant sites of DNA methylation in the genomes of higher eukaryotes. We wished to test whether the C-to-U deamination activity of the CG specific, commercially available C5-MTase M.SssI can be harnessed for this goal. 

Results of previous studies on M.SssI-mediated cytosine deamination were conflicting. Using antibiotic resistance reversion assays very similar to that used in the present study, two groups demonstrated M.SssI-catalyzed C-to-U conversion [7,10]. A third group used a different genetic reversion assay, and did not find evidence for cytosine deamination by either M.SssI or M.HpaII [4]. Finally, Metivier et al. reported that M.SssI can deaminate 5-methylcytosine as well as cytosine [13].

 Here we showed that M.SssI can catalyze deamination of cytosines located in CG dinucleotides in double-stranded DNA if the methyl donor SAM is omitted from the reaction (Figure 1). The deamination rate could be increased by adding 5’-amino-5’-deoxyadenosine to the reaction (Figure 5). These results confirmed conclusions of three of the previous studies [7,10,13]. Because of the different experimental conditions (pH, incubation time and temperature, presence or absence of Mg^2+^) the quantitative results of this work and of previous studies [7,10] are difficult to compare. The mean reversion frequency we obtained for M.SssI-catalyzed deamination under our standard *in vitro* conditions (4h incubation at 30°C, pH8.5, etc., see Materials and Methods), was ~2.7 x 10^-4^, which is ~10-fold higher than the frequency observed by Bandaru et al. after ~4 h incubation at 37°C [10]. Zingg et al. reported a revertant frequency of ~10^-4^, but this value was determined after 16 h of incubation at 37°C [7], and it is unclear whether the enzyme stayed functional for such extended period of incubation. It is possible that the faster reversion rates we observed were the consequence of incubation at 30°C, at which temperature, in our hands, M.SssI had higher MTase activity than at the widely used 37°C. 

In our system the combined action of M.SssI and 250 µM 5’-amino-5’-deoxyadenosine resulted in an elevated reversion frequency of 3.3 x 10^-3^, which is very close to the value obtained by the Jones group using 16h incubation [7]. 

 Besides studying this side activity of M.SssI *in vitro*, we wished to test whether the enzyme can be used to deaminate cytosines *in vivo*, in the presence of SAM. To this end, we constructed two mutants, which carried the F17S or the G19D replacement in the presumed SAM binding pocket. The rationale of creating these mutations was to weaken binding of the methyl donor SAM to the MTase and thus mimic conditions of limiting SAM in vivo, at physiological SAM concentrations. This approach, previously applied to HpaII MTase [30], proved successful also for M.SssI: we could detect elevated cytosine deamination rate with the G19D mutant in *E. coli* cells (Figure 4). We studied cytosine deamination in two types of *E. coli* cells. In one of the hosts, the *sssIM* allele and the reversion target KanS gene were on separate plasmids. The other host carried the KanS gene on the chromosome. Another difference between the two arrangements was that in the first one the *sssIM* alleles were on a low, whereas in the second one on a high copy number plasmid, presumably resulting, in the two hosts, in very different intracellular M.SssI concentrations upon arabinose induction. The G19D variant increased reversion to Kn^R^ phenotype in both types of Ung^-^ hosts, whereas the WT enzyme or the F17S mutant had no effect (Figure 4A). The increase in the rate of C-to-U conversion was also demonstrated by fluctuation test (Figure 4B). M.SssI(G19D) increased deamination rate also in the Ung^*+*^ control hosts (Figure 4), suggesting that the MTase can block uracil-DNA glycosylase mediated excision of the uracil from the U:G base. 

The very low residual MTase activity of the mutant enzymes (Figures 2B and 3) and the increased cytosine deamination rate observed with the G19D mutant (Figure 4) were consistent with the envisioned effects of impaired SAM binding. Other C5-MTases carrying replacements in conserved motif I showed similar loss of MTase activity [30,37,38]. The F17S and G19D substitutions of M.SssI correspond to the F38S and G40D replacements in M.HpaII (Figure 2A). Both residues (FxGxG) are strictly conserved in C5-MTases and participate in forming the SAM binding pocket probably in all C5-MTases [1,28]. For M.HpaII as well as for M.SssI, replacement of the glycine was more effective in promoting the cytosine deaminase activity ([30] and this work). 

 The data reported here show that the G19D mutant of M.SssI can be used as a CG-specific cytosine deaminase *in vivo*. This result raises the possibility of using G19D as a CG-specific targetable cytosine deaminase to induce C-to-T transitions at pre-determined CG sites *in vivo*. In the envisaged application, which has conceptually much in common with targeted DNA methylation [39,40], M.SssI(G19D) will be genetically or chemically fused to a targeting domain such as a zinc finger protein or a triple helix forming oligonucleotide designed to sequence-specifically bind to the DNA in the vicinity of the targeted CG site. It is expected that the targeted cytosine will be preferentially deaminated. It is worth mentioning that G19D has two features that can be beneficial for the planned application. The relatively low cytosine deaminase activity can be an advantage for achieving high targeting specificity as has been shown for targeted DNA methylation [41,42]. The high level of insensitivity to uracil excision repair observed in *E. coli* Ung^+^ hosts can be important for preventing repair of the pre-mutagenic U:G mismatch generated by the enzyme before conversion into a stable T:A mutant base pair. 

This work was initiated on the assumption that cytosine and 5-methylcytosine would show different sensitivities to M.SssI-catalyzed deamination. This notion was based on the similar reaction mechanisms of C5-MTase mediated and bisulfite mediated C-to-U deamination. In particular, both reactions are thought to produce the unstable 5,6-dihydrocytosine intermediate, which readily undergoes hydrolytic deamination [7,43]. In single-stranded DNA the rate of bisulfite-mediated conversion of C-to-U is ~50-fold higher than the rate of ^m5^C-to-T conversion [44]. This difference of reactivities forms the basis of bisulfite sequencing [35]. We compared the reactivities of cytosine and 5-methylcytosine to M.SssI catalyzed deamination in double-stranded DNA, and found conditions (presence of 5’-amino-5’-deoxyadenosine) where the difference between the reversion frequencies (and presumably between the deamination rates) was at least 100-fold (Figure 6). In light of the difference in the reactivities to bisulfite-mediated deamination [44], the difference between the rates of M.SssI-catalyzed conversions appears to be sufficient for reliable discrimination between unmethylated and C5-methylated cytosines. Unfortunately, the rate of M.SssI-catalyzed C-to-U conversion is too low to be a useful enzymatic alternative to the bisulfite reaction. However, it is possible that the enzyme can be “improved”, i. e. its cytosine deaminase side activity can be enhanced by directed enzyme evolution. Availability of the host strain ER2357 *ung*- constructed in this study offers a directed evolution strategy to select M.SssI variants with increased cytosine deaminase activity. In the envisioned selection scheme a plasmid library carrying *in vitro* mutagenized *sssIM* gene variants would be introduced into this host strain and pools of Kn^R^ revertants would be selected after a period of growth. The plasmid preparation isolated from the Kn^R^ culture would be mutagenized again and transformed into ER2357 *ung*-. We expect that multiple rounds of mutagenesis coupled with successively shorter growth periods will gradually enrich M.SssI variants with higher cytosine deamination activity. 

In summary, our results show that M.SssI can catalyze deamination of cytosines in CG dinucleotides in double-stranded DNA. Under the same *in vitro* conditions, M.SssI-catalyzed deamination of 5-methylcytosines was not detectable. Although the difference between the reactivities of C and m5C suggest that M.SssI could be used to determine the methylation status of cytosines in the epigenetically important CG sequence context, the slow rate of M.SssI-catalyzed C to U deamination makes this reaction, at present, impractical as an enzymatic alternative to the bisulfite reaction. We have shown that the G19D mutant of MSssI can catalyze C to U deamination *in vivo*, in *E. coli*, in the presence of SAM, even if the host is proficient in uracil excision repair. Thus, M.SssI(G19D) can function as a sequence-specific mutator that converts CG dinucleotides to TG. 

## Supporting Information

Figure S1 **Detection of C to U and ^m5^C to T change as a result of deamination.** Unmethylated or *in* *vivo* M.SssI-methylated pUP41 was incubated with M.SssI in the absence of SAM and transformed into *E. coli* ung or ung+ host to detect Kn^R^ revertants . Plasmids isolated from Kn^R^ revertants were digested with SmaI or MvaI. Agarose gel electrophoresis of the digested plasmids.
1) pUP41, untreated.
2) pUP41 incubated with M.SssI *in* *vitro*.
3) pUP41 methylated by M.SssI *in* *vivo*, and subsequently incubated with M.SssI *in* *vitro*.
M) Size marker (1 kb ladder, Fermentas).
Deamination reactions did not contain SAM.
There are 2 SmaI sites and 13 MvaI sites in pUP41. Disappearance of a SmaI site (CCCGGG) and appearance of a new MvaI site (CCWGG) indicates C to T change in the middle of the SmaI site (underlined). One of the new MvaI fragments (1249 bp), is marked by an arrow, and the disappearing SmaI (687 bp) fragment is marked by asterisk. Faint bands in samples 2 and 3 co-migrating with fragments of the untreated DNA (sample 1) probably indicate mixed plasmid population resulting from incomplete plasmid segregation.
(TIF)
