# Supplementary figures and images for: Cytosine-to-Uracil Deamination by SssI DNA Methyltransferase
Source: PLoS One. 2013 Oct 21;8(10):e79003. doi: 10.1371/journal.pone.0079003 (PMC3804486; doi:10.1371/journal.pone.0079003)

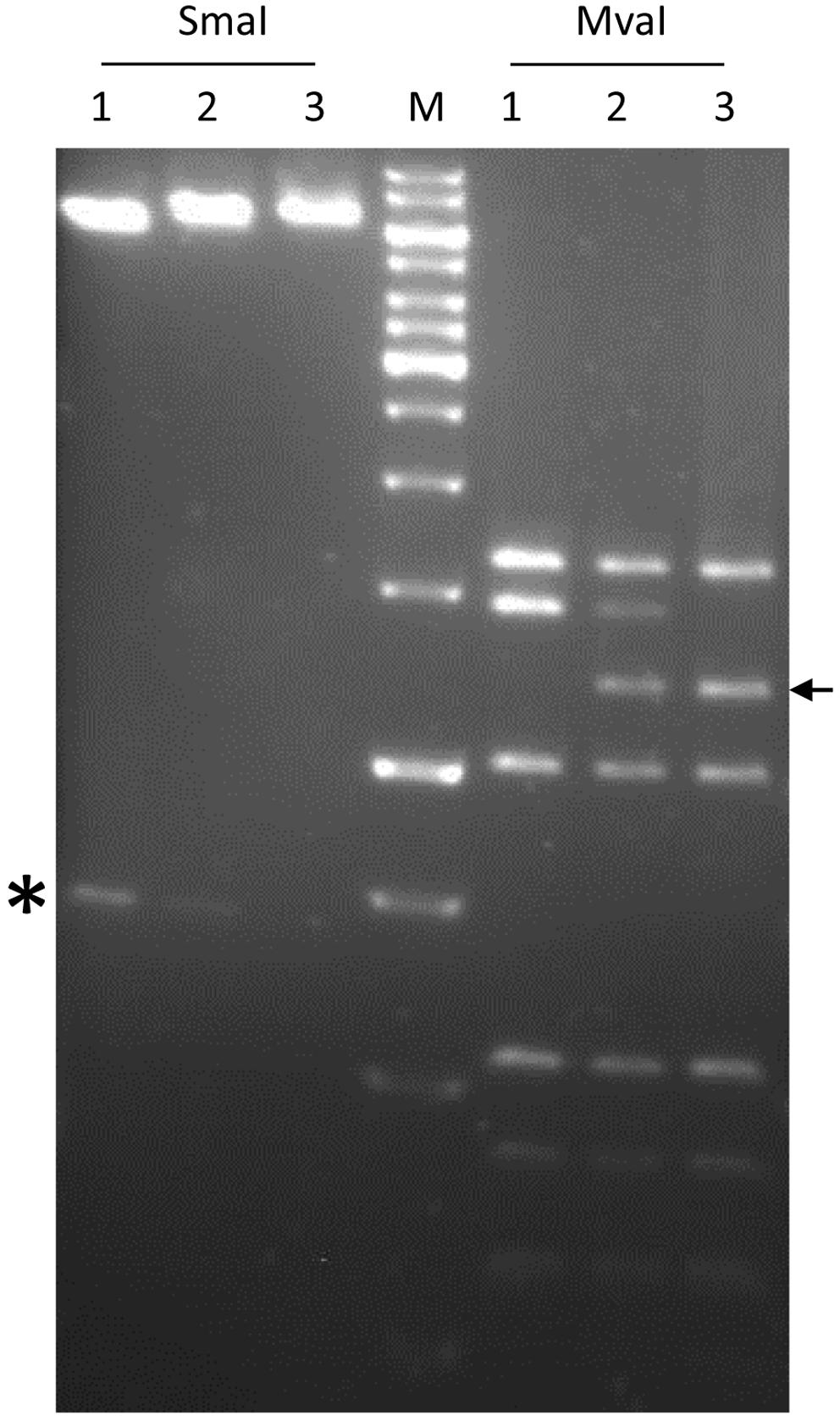

Supplement: Figure S1 — Detection of C to U and m5C to T change as a result of deamination. Unmethylated or in vivo M.SssI-methylated pUP41 was incubated with M.SssI in the absence of SAM and transformed into E. coli ung or ung+ host to detect KnR revertants . Plasmids isolated from KnR revertants were digested with SmaI or MvaI. Agarose gel electrophoresis of the digested plasmids. 1) pUP41, untreated. 2) pUP41 incubated with M.SssI in vitro. 3) pUP41 methylated by M.SssI in vivo, and subsequently incubated with M.SssI in vitro. M) Size marker (1 kb ladder, Fermentas). Deamination reactions did not contain SAM. There are 2 SmaI sites and 13 MvaI sites in pUP41. Disappearance of a SmaI site (CCCGGG) and appearance of a new MvaI site (CCWGG) indicates C to T change in the middle of the SmaI site (underlined). One of the new MvaI fragments (1249 bp), is marked by an arrow, and the disappearing SmaI (687 bp) fragment is marked by asterisk. Faint bands in samples 2 and 3 co-migrating with fragments of the untreated DNA (sample 1) probably indicate mixed plasmid population resulting from incomplete plasmid segregation. (TIF) [file pone.0079003.s001.tif]
